# myoActivation® for Chronic Pain Care That Changes the Biotensegral Properties of the Human Body: A Motion Laboratory Case Report

**DOI:** 10.7759/cureus.105076

**Published:** 2026-03-11

**Authors:** Jessica Luo, Lise Leveille, Nicholas West, Farah T Azim, Tim Bhatnagar, Gillian R Lauder

**Affiliations:** 1 Faculty of Science, University of British Columbia, Vancouver, CAN; 2 Research Institute, BC Children’s Hospital, Vancouver, CAN; 3 Department of Orthopaedics, University of British Columbia, Vancouver, CAN; 4 The Motion Lab, Sunny Hill Centre at BC Children's Hospital, Vancouver, CAN; 5 School of Biomedical Engineering, University of British Columbia, Vancouver, CAN; 6 Department of Pediatrics, Faculty of Medicine, University of British Columbia, Vancouver, CAN; 7 Department of Department of Anesthesiology, Pharmacology and Therapeutics, University of British Columbia, Vancouver, CAN

**Keywords:** biotensegrity, chronic pain, fascia, motion capture, myoactivation, myofascial dysfunction

## Abstract

The human body is a biotensegral structure capable of bending and twisting in multiple directions as a functional, integrated movement unit, while maintaining balance and an ability to return to any pre-movement position. Myofascial dysfunction caused by soft tissue stiffness - muscles in sustained contraction, tethered skin, scars, or anomalous fascial tension - can affect the whole fascial continuum, leading to mobility impairment and chronic pain. Myofascial dysfunction is not just a local phenomenon, as the range of motion at one skeletal articulation may affect the whole system. myoActivation® is a structured assessment and therapeutic process designed to recognize and manage myofascial dysfunction and chronic pain. The Complex Pain Service at British Columbia’s Children’s Hospital utilizes myoActivation® as a tool in the interdisciplinary care of adolescents with a myofascial component to their chronic pain. This case report illustrates how myoActivation® can improve movements at distal joints, not just those that were predicted to change based on the soft tissues treated. A 16-year-old female with a complex surgical history for embryonal rhabdomyosarcoma of the bladder presented with a one-year history of right-sided chronic low back pain. Imaging and bloodwork investigations were unremarkable. On assessment by the Complex Pain Service, signs of myofascial dysfunction were identified, including active trigger points in skeletal muscles, fascial restrictions, and scar involvement. Targeted muscle activation, as well as scar and fascial release, were performed over a course of four myoActivation® sessions. Objective Clinical Motion Analysis using motion capture technology demonstrated improved maximum range of motion and/or speed of motion in 55% of her movement tests after the initial myoActivation® session and in 100% of assessed movement tests by the final session. Improvements were observed not only in expected areas but also in distal joints, supporting the biotensegral model of human movement. The patient reported reduced pain, increased engagement in daily activities, and full functional recovery without the need for ongoing analgesia. This case demonstrates that myoActivation® impacts the human body’s biotensegral properties. The improvements observed across both proximal and distal movement patterns underscore the importance of considering the biotensegral nature of the body in medicine.

## Introduction

Myofascial dysfunction is often not recognized as an etiological component of mobility impairment and chronic pain presentations. Classically, myofascial dysfunction is poorly defined. For the purposes of this case report, myofascial dysfunction is the tightness and distortion of soft tissues related to active trigger points of skeletal muscles in sustained contraction, dense/thickened fascia, tethered skin, scars, or a combination of these issues [1,2].

Myofascial dysfunction can be understood using principles of tensegrity, an architectural principle in which structural integrity is maintained by continuous balance of tension and compression resistance forces, and biotensegrity; the application of tensegral principles to biological structures, such as the human body [3]. Myofascial dysfunction usually develops in response to trauma, injury, surgery, acute or repetitive strain, recurring soft tissue microtrauma, incorrect posture, muscle overuse, trigger point activity in other muscles, nutritional deficiencies, inflammatory processes, and metabolic derangements [4-6]. Active muscle trigger points involve a wide range of pathological changes [7,8] that promote sustained contraction of muscle fibers [9]. This creates a palpable, painful trigger point within a taut band and shortened muscle [10], commonly responsible for pain [4] and alterations in the patterns of whole-body movements [11]. Palpable, painful fascial trigger points can be caused by fascial adaptation to repeated stress, tension, or trauma, which may result in increased fascial density or thickness and tightness. These are often located near active muscle trigger points, which can incite fascial triggers and vice versa. The dynamic symmetry of the biotensegral body can be interrupted by scars, both locally and at distant sites, through pathological adhesions and restricted fascial chains [12]. Even small scars or scars, which appear to look normal, can have a myofascial impact [10,13].

myoActivation® is a systematic assessment and therapeutic process used in the management of mobility impairment and pain secondary to myofascial dysfunction. The process of myoActivation® has been previously described [12,14] and is included in Appendix 1. Preliminary findings suggest that myoActivation® has an immediate and sustained beneficial effect on pain, range of motion (ROM), and flexibility [14-17].

Clinical Motion Analysis (CMA) is the systematic study of human motion using observed and measured body movements, body mechanics, and the activity of muscles. The Motion Lab (TML) at BCCH’s Sunny Hill Health Centre is a state-of-the-art facility for conducting CMA and pedobarography. During myoActivation® movement tests, TML can facilitate the capture of joint kinematics and kinetics, as well as weight distribution data, which can be used to characterize aspects such as ROM, joint loads, and fluidity of movement. The methods for generating models for kinematic and kinetic analysis are described in a pilot research study of myoActivation® with TML [15]. This case report describes the improvements observed at distant sites from treatment in one participant from the pilot study, which highlights the biotensegral principle.

## Case presentation

Ethical approval was originally obtained for the pilot study (#H20-00463, approval date March 9, 2022) [15]. The patient provided written informed consent to participate in the pilot study. Further consent was also obtained from the patient specifically for this case report. This manuscript was prepared in accordance with the CAse REports (CARE) guidelines.

The pilot study

In the pilot study [15], five adolescent patients performed the standard set of Biomechanical Assessment and Symmetry Evaluation (BASE) movement tests [12] during each of their myoActivation® sessions in TML. CMA was used to record the movement tests performed before the first myoActivation® treatment session (baseline), immediately after the first treatment session (initial), and after they completed their last session (final). Meaningful changes in ROM and speed of motion were demonstrated after a single myoActivation® treatment session, while further improvements were shown over the course of serial treatments. The myoActivation® clinician predicted some of the improvements based on which soft tissues had been treated. However, there were improvements in other movements that had not been predicted to change in some of these cases. This case report demonstrates that myoActivation® impacts the human body’s biotensegral properties with objective improvements in movement at distal joints, not just of movements that were predicted to change.

The patient

A 16-year-old, 60 kg, female was referred to the BCCH Complex Pain Service (CPS) for chronic low back pain (CLBP) of one year in duration. She had a past medical history of embryonal rhabdomyosarcoma of the bladder, which was treated at age 2 with chemotherapy, radical cystectomy, and creation of a neobladder and Mitrofanoff stoma. She did not receive radiotherapy. She had been under surveillance with her oncology team since diagnosis and had not had tumor recurrence. Her CLBP began one year previously when she was bending over to retrieve an item from a low-level shelf; she felt a twisting motion in her lower back with this movement. She first noticed a cramping pain in the right side of her lower back. This pain gradually increased over the coming months to the point where she was having shooting pains over the right side of her lower back. The pain did not radiate down her legs. It was not associated with leg weakness. There was no saddle anesthesia. She denied any unexplained fevers, rashes, weight loss, pins and needles, skin numbness, joint swellings, bowel issues, sacral numbness, skin hypersensitivity, or changes in skin color or temperature. She described the pain as intermittent. She stated that the average intensity of pain in her right lower back was 7/10, and it predominantly had an aching quality. She stated that she had minimal pain when she woke up in the morning, but toward the middle of her day, she would feel increasing pain intensity. Her pain-provoking factors included bending forward and walking for longer than 10 minutes. The patient reported that acetaminophen and ibuprofen provided some pain relief. The exact dosage and frequency were not available as this information was patient-reported. She had not tried any other analgesic medications or supplements.

Impact of pain

Her CLBP impacted her schoolwork, reporting that she was too uncomfortable to do schoolwork for up to 30 days in the three months prior to the time of her initial CPS assessment. Her sleep and appetite were not impacted by her pain. Persistence of her back pain did, at times, make her angry and frustrated. She reported that she felt anxious that movement would worsen her back pain, such that she avoided participation in physical education classes and had forgone tennis lessons, which previously brought her joy. When asked, she denied any sadness, depression, or episodes of self-harm.

Previous investigations and remedies

She reported that she had visited the emergency department multiple times for assessment and management of her CLBP. She had undergone multiple physical examinations, all of which were reported to be normal. She was assessed by oncology, urology, pediatrics, and orthopedics. Previous classical examinations, imaging (X-ray and MRI), and bloodwork revealed no remedial cause for her pain. The patient previously had significant pelvic and suprapubic abdominal pain, which resolved with the initiation of oral contraceptive therapy under the care of gynecology. Prior to her CPS referral, she was already undergoing acupuncture and chiropractic care. She had not been involved with physiotherapy, massage therapy, or psychology support.

Timeline of Lifetime Trauma

She had not been involved in any motor vehicle accidents. At age 10, during a trampoline injury, she had a right elbow dislocation and fracture, which was managed conservatively. She had no other significant injuries. When asked about the greatest physical impact on her body in her life, she stated it was her trampoline injury, but her mother believed that it was her surgeries and bladder reconstruction for her rhabdomyosarcoma. The classic medical examination was normal.

myoActivation® examination

On initial myoActivation® examination, she had an uneven distribution of weight on her feet. There were no postural anomalies except a medial deviation of the left knee. She had four abdominal scars, two chest scars from bilateral post-insertion and removal, and a scar on her right neck for the proximal port site. Extension Arms Raised (EAR) was the most restricted and painful BASE test, indicating that the paraspinal muscles were playing a predominant role in her CLBP. On palpation, the right paraspinal was taut and painful to palpation. Other muscles in sustained contraction included the left iliopsoas and left quadratus lumborum (QL). Fascial tension was noted at the bilateral iliac crests, left pubic fascia, and coccyx.

Treatment

The combination of the history and examination confirmed that there was a myofascial component to her pain profile. She was recommended conservative management initially; this is based on the 3P (Physiotherapy and massage, Psychology, and Pharmacology) approach, designed to help youth living with chronic pain achieve a primary goal of change from a pain-centered life to a function-centered life. She was started on magnesium bisglycinate, Vitamin D3, Vitamin K2, and Omega 3 (MgBis/K2/D3/omega 3) supplements designed to promote relaxation of muscles in sustained contraction and support an anti-inflammatory environment (see Appendix 2). Written myoActivation® information was given to the family. One month later, repeat examination revealed ongoing myofascial dysfunction, and it was deemed appropriate to proceed with myoActivation® [12]. Written consent for myoActivation® treatment was obtained. The patient underwent four sessions of myoActivation® with different muscles activated and scars and fascia released in each sequential session (Table 1).

**Table 1:** myoActivation® treatment details: muscles activated, scars and fascia released for all myoActivation® sessions. EAR, extension arms raised; EAD, extension arms down; TML, The Motion Lab CMA recording session

Session	Weeks after initial session + location	Most restricted or painful BASE test	Muscles activated	Scars released	Areas where fascia in tension released
1	Initial, TML	EAR	Right paraspinal	None	Left iliac crest, coccyx
2	+ 1 week, clinic	EAD	None	Abdominal scars × 4	Coccyx, left pubic fascia
3	+ 1 week, clinic	None	Bilateral paraspinals	Abdominal scars × 4	None
4	+ 3 weeks, TML	EAR and EAD	Right paraspinal, left iliopsoas, left vastus medialis	Bilateral port scars, left neck scar	Linea alba above umbilicus

Outcomes

Changes in measured maximum ROM (maxROM) and maximum angular speed to maxROM (speedROM) follow our previous paper [15]; changes were considered clinically significant if the change due to intervention resulted in an increase of 5° or 5°/s, respectively. The clinician predicted changes in movement tests were based on maxROM only, since speedROM could not be predicted. Based on objective CMA data, we found evidence of improved maxROM in 6/11 (55%) of the movement tests for the initial myoActivation® session; two (EAR, LA-L) were movement tests that the clinician had predicted would change after the initial myoActivation® session, four (squat arms raised (SAR), squat arms down (SAD), and single leg squat (SLS)) were movement tests that had not been predicted by the physician, and one (flexion arms down (FAD)) test did not show a significant increase (Table 2). When comparing data from baseline to final TML assessment, CMA found objective evidence of improved maxROM and/or speedROM in 11/11 (100%) movement tests (Table 3, Video 1).

**Table 2:** Objectively measured changes in movement tests for the initial myoActivation® session in The Motion Lab. EAR, extension arms raised; EAD, extension arms down; FAD, flexion arms down; HE, hip extension; HF, hip flexion; L, left; LA, lateral arch; maxROM, maximum range of motion; R, right; SAD, squat arms down; SAR, squat arms raised; SLS, single leg squat; speedROM, maximum angular speed to maxROM; TT, torso twist; TWRP-E, thorax with respect to pelvis - extension; TWRP-F, thorax with respect to pelvis - flexion; TWRP-R, thorax with respect to pelvis - rotation Improved maxROM and improved speedROM: “yes/no” indicators were selected as “yes” if two joints were analyzed for a movement and either one or both demonstrated improvement.

Movement tests			Objectively measured changes in kinematic data			
All	Predicted to change	Observed change	Improved maxROM	Increase in maxROM (bold indicates > 5°)	Improved speedROM	Increase in speedROM (bold indicates > 5°/s)
EAR	Yes	Yes	Yes	TWRP-E +13, HE -3	Yes	TWRP-E +7, HE 0
EAD	No	No	No	TWRP-E -5, HE 0	Yes	TWRP-E +9, HE +4
FAD	Yes	Yes	No	TWRP-F +2, HF +1	Yes	TWRP-F +13, HF +4
SAR	No	No	Yes	TWRP-F -3, HF +8	No	TWRP-F -6, HF -12
SAD	No	No	Yes	TWRP-F +1, HF +5	No	TWRP-F +4, HF -10
LA-L	Yes	Yes	Yes	L +6	No	L +3
LA-R	No	Yes	Yes	R +9	Yes	R +5
TT-L	No	No	No	TWRP-R +3	Yes	TWRP-R +15
TT-R	No	No	No	TWRP-R +4	No	TWRP-R 0
SLS-L	No	No	No	TWRP-F -3, HF -4	No	TWRP-F +4, HF +2
SLS-R	No	No	Yes	TWRP-F -1, HF +11	Yes	TWRP-F +1, HF +19

**Table 3:** Objectively measured changes in movement tests from baseline to the final Motion Lab analysis. EAR, extension arms raised; EAD, extension arms down; FAD, flexion arms down; HE, hip extension; HF, hip flexion; L, left; LA, lateral arch; maxROM, maximum range of motion; R, right; SAD, squat arms down; SAR, squat arms raised; SLS, single leg squat; speedROM, maximum angular speed to maxROM; TT, torso twist; TWRP-E, thorax with respect to pelvis - extension; TWRP-F, thorax with respect to pelvis - flexion; TWRP-R, thorax with respect to pelvis - rotation Improved maxROM and improved speedROM: “yes/no” indicators identified “yes” if two joints were analyzed and either one or both demonstrated improvement beyond the threshold.

Movement tests	Objectively measured changes in kinematic data			
	Improved maxROM?	Increase in maxROM (bold indicates > 5°)	Improved speedROM?	Increase in speedROM (bold indicates > 5°/s)
EAR	Yes	TWRP-E +7, HE +9	Yes	TWRP-E +5, HE +4
EAD	Yes	TWRP-E +3, HE +10	Yes	TWRP-E +14, HE +8
FAD	Yes	TWRP-F +12, HF -14	Yes	TWRP-F +16, HF -6
SAD	Yes	TWRP-F +14, HF -2	Yes	TWRP-F +10, HF +7
SAR	Yes	TWRP-F +16, HF +2	Yes	TWRP-F +9, HF -1
LA-L	Yes	L +9	No	L +4
LA-R	Yes	R +8	Yes	R +10
TT-L	Yes	TWRP-R +5	Yes	TWRP-R +11
TT-R	Yes	TWRP-R +7	Yes	TWRP-R +15
SLS-L	Yes	TWRP-F +19, HF -9	Yes	TWRP-F +15, HF +13
SLS-R	Yes	TWRP-F +6, HF +18	Yes	TWRP-F +11, HF +7

**Video 1:** Changes in movement tests from baseline to the final Motion Lab analysis.

In addition to the anticipated changes observed between the baseline and final TML assessments, significant improvements were also noted at the knee and ankle joints. The EAR movement test showed increased ankle dorsiflexion by 7.2° on the right and 7.1° on the left. During the EAD movement test, right ankle dorsiflexion improved by 5.7° and right knee flexion improved by 5.8°. For the SLS test on the left, maxROM increased by 7.0° in left knee rotation.

One month after the patient’s final myoActivation® session, she reported less pain and being much more engaged with academic and social activities. At the time of discharge, 11 months after her initial CPS assessment, the patient had no reported CLBP, was able to function physically with no restrictions or pain, and was attending school full-time. She did not need prescribed analgesic medications, nor any over-the-counter medications, and was advised to slowly wean MgBis/K2/D3/omega 3.

## Discussion

**Figure 1:**
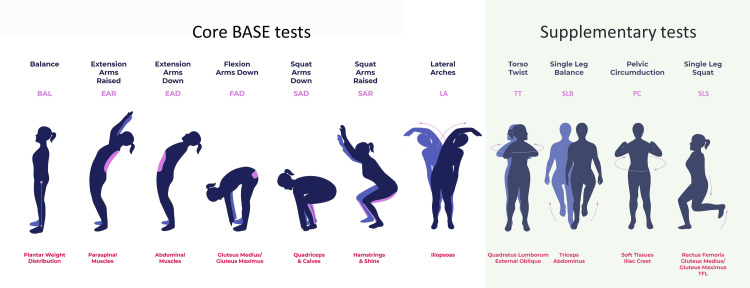
The seven core Biomechanical Assessment and Symmetry Evaluation (BASE) movement tests and additional basic movement tests that are used in myoActivation® assessment. The seven core tests include (left to right): balance (BAL), extension arms raised (EAR), extension arms down (EAD), flexion arms down (FAD), squat arms down (SAD), squat arms raised (SAR), and lateral arches (LA). The related tissues are described below each movement demonstration, in red text. Originally published by Bhatnagar et al. [15]. Image credits: Gillian R. Lauder with illustrations by Shona Massey (using Adobe Illustrator (Adobe Inc., San Jose, CA)).

The pilot study involving this case demonstrated objective evidence of improved ROM for movements that were predicted to change after myoActivation® using CMA [15]. According to the patient’s history and BASE movement tests (Figure 1), the clinician expected changes related to the sites of treatment, but not necessarily at distal sites. Improvements in mobility of the thoracic-pelvic joints were predicted; however, improvements were also found at other sites distal to the site of treated soft tissues, such as at the knee and ankle joints. This patient demonstrated increased lower limb ROM across multiple joints and movement tests over the course of myoActivation® treatment. Notable gains were observed during the SLS tests, suggesting enhanced mobility and control in dynamic, weight-bearing movement. It is possible that an increased ROM was observed at the knee and ankle joints to accommodate a change in the center of mass, as the hips extend further for EAD and EAR, or that the patient’s hip joint was functioning with more ease (i.e., from the myoActivation® therapy release), allowing the knee and ankle joints to also move more easily. It is postulated that a component of the distal site changes reflects the changes in the biotensegral properties of the myofascial tissues.

Tensegrity, biotensegrity, and myofascial dysfunction

Tensegrity describes structures that balance isolated compression elements with continuous tension, creating stability, adaptability, and energy efficiency. In this system, struts resist compression while cables resist expansion, and together they form a lightweight yet resilient framework in which each part influences the whole. Biotensegrity applies these principles to biology, where bones act as compression elements and soft tissues (muscles, fascia, and skin) provide tension. This interconnected design allows the body to function as a unified system that stabilizes, adapts to strain, stores, and releases energy efficiently. Each part contributes to the whole, meaning movement or tension in one area can affect even distant regions of the body [3,18].

The patient in this case may have developed myofascial dysfunction over many years in response to a combination of her previous surgeries and bladder reconstruction for rhabdomyosarcoma, as well as other factors such as the patient’s abdominal surgical scars and chest scars from previous port sites for chemotherapy. Each contributor to myofascial dysfunction (active muscle triggers, fascial density, skin tethering, or scars) involves important pathophysiological changes that impact chronic pain perception and the biotensegral properties of the body. myoActivation® helped to improve asymmetries, postural anomalies, limitation in ROM, and speed in the ability to perform movements in this patient.

Active muscle trigger points

Active muscular trigger points are irritable nodules, located predominantly near the motor end plates in taut bands of skeletal muscle [9]. Active muscle triggers are associated with failure of the calcium pump, secondary to ATP deficiency, where myosin and actin filaments remain in a maximally interconnected closed rigor complex. This results in a shortened muscle, muscle stiffness, restricted ROM, sensory changes, and pain, which is either a localized palpable, painful trigger point, referred pain, or distant site pain [5,19]. These active muscle trigger points and extra muscular connective tissue changes cause changed muscle activation patterns with alterations in the patterns of whole-body movements [11]. In this case, at the initial myoActivation® session, EAR was the most restricted and painful BASE test; therefore, the right paraspinal muscles were activated with release of the coccygeal and left iliac crest fascia.

Fascia

myoActivation® principles recognize the influence of fascia on the biotensegral system. Addressing fascial tension in this patient’s myoActivation® course may have supported the changes seen at local and distal articulations. The Fascia Nomenclature Committee defines the fascial system as consisting of “the three‐dimensional continuum of soft, collagen-containing, loose and dense fibrous connective tissues that permeate the body […] The fascial system surrounds, interweaves between, and interpenetrates all organs, muscles, bones, and nerve fibers, endowing the body with a functional structure and providing an environment that enables all body systems to operate in an integrated manner” [20].

Different regions of fascia have classically been named according to their distinct locations; for example, the pubic fascia or sternoclavicular fascia. Based on the above definition, fascia should not be thought of as separate entities - they are rather a single continuous entity arranged in several layers with each layer characterized by a different direction and thickness. In addition, muscles and fascia exist in well-described myofascial chains [21]. These myofascial chains enable movement in different directions while continually providing information on balance, stability, and mobility. The myofascial chains often have an opposing chain to help achieve balance within the biotensegral system; for example, a posterior myofascial chain couples with an anterior myofascial chain. These chains may well help to explain how myofascial release at distant sites (or opposite sides of the body) resolves coupled pain presentations. For example, as in this case release of soft tissue around the pelvis and abdomen improved joint movements at the knee and ankle.

Not only does fascia provide support, protection, and structure, but it is also a sliding and gliding mechanism that enables movement between adjacent tissues. Hyaluronan, an extremely hydrophilic, highly viscous substance found throughout fascia, serves as a lubricant for these slide and glide properties. There are over 100 million receptors in the fascial system [22], making fascia one of the largest sensory organs of the body, constantly communicating and exchanging proprioceptive, interoceptive, neurosensory, and nociceptive information [23,24]. The viscoelastic properties of fascia change in response to physical forces. Physical activities, healthy movement patterns, stretching, and exercise promote healthy fascial plasticity that results in improved flexibility, better tissue resilience, and enhanced performance. This adaptive nature of fascia, as well as alterations in hyaluronic acid content in response to different stimuli, is the reason for the improved pliability and mobility of fascia after manual therapy or soft tissue release [25].

Fascial trigger points

Fascial tissues subjected to repeated stress, asymmetrical tension, poor posture, sedentary status, abnormal movement patterns, injury, or surgery adapt by increasing collagen production, altering fiber arrangement, reducing elasticity, and altering their mechanical properties [26]. Although the fascia released in this case was mostly located around the lower abdomen, iliac crest, coccyx, and pubis, the resting muscle tone may have also been significantly influenced by changes in fascial tone [27] due to enhanced myofibroblast density and activity in the muscle perimysium [28]. Myofibroblasts develop from regular fascial fibroblasts under the influence of mechanical strain, sympathetic stimulation, and cytokines such as TGF-β1 [28,29]. Nerves and blood vessels pass through the fascial network so that fascial density and fascial trigger points can cause compression of these structures, thereby increasing myofascial dysfunction [29]. Fascial density results in altered proprioception, biotensegral distortions, changed interoception, and myofascial pain [30-32].

Scars

The scar release process of myoActivation® may have played a role in the overall improvements in this case [13,33,34]. Scars display characteristics that differ from normal skin. They may be thickened, dense, rough, have compromised elasticity and mobility, altered or abnormal neural functioning, and discoloration [35,36]. These scars can create adhesions between organs after injuries, infections, or surgeries. Due to the high concentration of nerve endings in the visceral fascia, scars can sensitize the surrounding tissues and trigger viscerosomatic reflexes. During the scar healing process, fascial layers and soft tissues may adhere to one another, causing tensions that bridge from the scar and surrounding skin to deeper soft tissue layers and associated myofascial planes [37]. The human body is constantly adapting to the stresses imposed on it, and forces disseminated throughout the body are impacted by injury or scars. The abdominal scars in this patient contributed to her CLBP; myoActivation® treatment may have supported release of the anterior and posterior myofascial chains.

Limitations

The evidence in this case report is based on a single participant. Further limitations for this case report are presented in the initial report of our pilot study (e.g., the negative kinematic changes observed could potentially be due to marker placement error) [15]. More cases are required to demonstrate the changes at distal articulations, remote from the locations of needling and the perceived site of pain.

## Conclusions

This case report utilized motion capture technology to provide further evidence of beneficial changes in ROM in an adolescent patient undergoing myoActivation® for CLBP related to myofascial dysfunction. The patient’s improvements in movement tests were noted in areas beyond those predicted to change based on the pre-treatment myoActivation® assessment. This case demonstrates the important concept of biotensegrity, highlighting how fascia, skin, scars, and muscles in sustained contraction combine to influence the whole body. Other clinicians should consider the role of myofascial dysfunction in complex pain presentations. myoActivation® may provide clinicians with an effective, low-cost, drug-free strategy for patients diagnosed with myofascial dysfunction and pain.

## Appendices

Appendix 1: the myoActivation® process

myoActivation® Summary

Myofascial dysfunction occurs in response to past injuries or surgeries, where the soft tissue injuries from these past traumas result in scars, skin tethering, muscles in sustained contraction, or densified and tight fascial tissues. These soft tissue impacts can occur either in isolation or as a combination of all these factors. Myofascial dysfunction is commonly overlooked as a contributing etiological factor in chronic pain presentations. myoActivation® is a unique structured system of assessment and treatment designed to reduce myofascial components of chronic pain. A core principle of myoActivation® is that the site of pain is often not the source of pain due to the myofascial connections and biotensegral properties of the human body. myoActivation® assessment starts with a detailed injury history dating as far back in time as can be remembered. This Timeline of Lifetime Trauma (TiLT) includes the collective history of past motor vehicle accidents, fractures, sprains, falls, tailbone injuries, burns, bites, major and minor surgical procedures, and other scars. The myoActivation® practitioner then assesses posture and observes standardized functional movements called Biomechanical and Symmetry Evaluation (BASE) tests. The most restricted or painful BASE test directs the myoActivation® practitioner to the most likely tissues impacting myofascial dysfunction. After inspecting the identified area for skin tethering or scars, the clinician then examines the area for palpable pain points to identify dominant areas of myofascial tension. The practitioner amalgamates the information from the reported TiLT, posture assessment, BASE test assessment, and examination to direct care. The myoActivation® intervention is a needling technique utilizing fine-gauge hollow-bore, cutting-tip hypodermic needles with no injectate. Needling at the palpable pain points is designed to release myofascial tension. The BASE tests and palpation are repeated after each treatment (catenated cycles) to determine the next most dominant myofascial site that needs to be treated. The number of myoActivation® sessions required to loosen sources of myofascial dysfunction varies depending on each individual. myoActivation® sessions are usually spaced one to two weeks apart. Usually, four to eight myoActivation® sessions are required to unravel multiple sources of myofascial dysfunction in a longitudinal process, but more sessions may be required if there is an ongoing trigger for myofascial dysfunction, for example, a patient with a history of previous significant burns and scarring will likely need many more sessions for ongoing release of soft tissues. The BASE tests are improved first, then there are other regional tests for specific areas such as the neck, shoulder, or jaw. After treatment, immediate aftercare includes regular posture change to allow the treated tissues to recover. Long-term aftercare requires attention to increasing physical activity and strengthening soft tissues. Risks of myoActivation® include discomfort at the site of needling, bruising, dizziness, light-headedness, emotional responses, post-care fatigue, muscle aches, changing sensation of pain, and, very rarely, pneumothorax when needling over the chest wall. myoActivation® clinicians have observed, and patients report that treatment results in immediate improvement or changes in pain, flexibility, and ease of movement. Often, this is appreciated after the first session, but with multiple sources of myofascial dysfunction, this may not be realized until session 2 or 3 in this longitudinal process.

Assessment

Timeline of Lifetime Trauma (TiLT): The TiLT is essentially a detailed history from as far back as the patient can remember of any motor vehicle accidents, fractures, sprains, falls, tailbone injuries, major surgeries, minor surgeries, burns, bites, or other scars. The mechanism of each injury is sought to help elucidate which soft tissues may have been impacted. The associated healing process of any scar is essential to determine its significance in the pain presentation. Clinical experience suggests that infection during the healing process and injuries sustained at the youngest age appear to have a significant impact on myofascial dysfunction. An important enquiry in the myoActivation® history is to ask the patient what they consider to be their greatest physical trauma. Scars are particularly relevant even if they appear to look normal or are very small. These details help the myoActivation® clinician determine what soft tissue may have been injured and therefore where the patient may have myofascial dysfunction. For example, a scar in an area hidden by clothing may have never been found if the myoActivation® clinician did not ask and know these details. The TiLT is integrated with the subsequent examination findings to help determine their significance in the current chronic pain presentation.

Observation: The myoActivation® clinician observes the patient for asymmetry in gait, seated stance, or standing posture during the clinical encounter. As examples, a knee in flexion during the swing phase of walking may indicate that the ipsilateral hamstrings or lateral abdominal wall muscles are in sustained contraction, a seated stance where the patient sits with the feet internally rotated may indicate tightness in the medial fascial plane or the adductors are in sustained contraction, or, when standing pelvic rotation may indicate that the ipsilateral TFL, iliopsoas, and rectus femoris or contralateral gluteus medius are in sustained contraction. Initially, the patient is asked to identify the location of their perceived pain; this helps direct the examination and is used as an index for subsequent treatment effect. Where the patient identifies or indicates the perceived site of pain is rarely the tissue that is responsible for the true origin of pain; for example, lower quadrant abdominal pain may originate from an ipsilateral quadratus lumborum muscle in sustained contraction.

Postural assessment: The clinician observes the standing postural and notes if there are any differences between the right and left sides of the body reviewing; feet (e.g., pronated, elevated little toe, clawed toes), knees (e.g., hyperextended or hyperflexed), level of the hips, any pelvic rotation or tilt, shoulder height, as well as any shift or tilt of the torso and head position). As examples, hyperextended knees indicate that the quadriceps muscles are in sustained contraction, a head forward posture would indicate that the sternocleidomastoid, platysma, or anterior abdominal wall musculature was in sustained contraction, or that the sternal fascia is tight.

Core BASE tests: These tests are used to screen a patient’s body for the true origin of pain. BASE tests compartmentalize the true origin of pain to a defined anatomical region (Figure 1). First, the patient is asked about the perceived weight distribution on their feet (right = left, back, front, or central and outside inside or central). The talus has no muscular attachments, so it functions as a ball and socket joint around which the skeleton sways depending on the distribution of myofascial forces. In humans, the center of the body mass is normally located anterior to the S2 vertebrae. In an erect stance, if there is no significant anatomical postural distortion, the center of mass or gravity will be evenly distributed between the feet and over each plantar surface. Therefore, if one foot feels heavier than the other, then there is a shift of the center of mass or gravity toward that side of the body. For example, if weight is perceived to be more on the right foot, then there is likely contracted musculature in the right leg, “pulling” the pelvis to the right and shifting the center of mass to the right. One of the aims of the therapeutic component of myoActivation® is to change the perceived balance of weight on the feet to be more in equilibrium.

Identification of the area of greatest myofascial dysfunction: The subsequent tests are used to identify the most painful or restrictive BASE test. Administering these core BASE tests is a quick assessment. The most painful or restrictive BASE test identifies the tissues that are most likely contributors to the perceived pain. Even though the individual BASE tests are common human movements, the coordinated use of these movement tests to define anatomical areas that are the true origin of pain is unique. Each test defines a specific muscle group or body area, as shown in Figure 1. The most painful or restrictive test generally provides a clear indication of a starting point for treatment when a patient has multiple sites of pain or widespread pain. For example, if EAR is the most restricted or painful test for an individual patient, then the myoActivation® clinician will first inspect and examine the paraspinal region. The individual BASE tests are explained below:

- Extension arms raised (EAR): The patient is instructed to bend backward from the hips with their arms overhead. Wherever pain is perceived by the patient in this posture, the true source of pain originates in the paraspinal muscles.

- Extension arms down (EAD): The patient is instructed to arch backward from the hips with their arms down. Wherever pain is perceived by the patient in this posture, the true source of pain originates in the abdominal muscles.

- Flexion arms down (FAD): The patient is instructed to flex forward with straight knees and bend forward to wherever they can reach comfortably. The patient is questioned specifically about pain in the lower back. If pain is perceived in the low back in this posture, the true origin of pain is in the medial gluteus medius and/or gluteus maximus muscles.

- Squat arms down (SAD): The patient is instructed to squat with their arms by their side to where they can crouch comfortably. If a patient has a very restricted squat, their technique in performing the squat can be improved by instructing them to drive their buttocks backward. A deeper squat will invariably result due to increased pelvic rotation from this maneuver. Wherever pain is perceived by the patient in this posture, the true origin of pain is in the quadriceps or calf muscles. If the pain is perceived to be in the upper leg, then the quadriceps will be the pain source. If in the lower leg, then the gastrocnemius and/or soleus will be the source.

- Squat arms raised (SAR): The patient is instructed to squat with their arms overhead to where they can crouch comfortably. Wherever pain is perceived by the patient in this posture, the true origin of pain is in the hamstrings or tissues overlying the shin. If the pain is perceived to be in the upper leg, then the hamstrings will be the pain source. If the pain is in the lower leg, then the medial tibial fascia or soft tissues will be the source.

- Lateral arches: The patient is instructed to bend to the side (like a ballerina). Wherever pain is perceived by the patient in this posture, the true origin of pain is in one of the iliopsoas muscles.

- Inspection: The myoActivation® clinician inspects the region of the most painful or restricted BASE test for any asymmetrical features, tethered skin, skin creases, fascial lines of tension, or any scars, even tiny ones. For example, if SAR is the most restricted or painful BASE test with pain reported in the lower legs, doing that movement, then any scar on the shin will be significant, even if it is a tiny chicken pox scar or a seemingly insignificant scar from a childhood injury.

- Palpation: The technique of palpation requires a rolling motion to be used, applied using both thumbs or index fingertips simultaneously, on symmetrical tissues to compare right and left sides. Differences between right and left may be apparent by the patient’s physical reaction, the patient’s verbal report, and/or by sensory feedback to the examiner from digital pressure. The sensory feedback of the clinician improves with experience.

The goal in palpation of soft tissues is to identify increased density, which is painful to the patient and feels different to the clinician when comparing the same tissue on the other side. In most instances, when increased density of a soft tissue is identified, the patient will express or react to the noticeable increase in discomfort or pain associated with palpation of the abnormal tissue. The painful palpable pain points are very important as an indicator of a myofascial source of pain.

Therapeutic Technique

Needling technique: Needling is performed with fine-gauge (25-30 gauge) hollow-bore cutting-tip needles that are similar to the needles used for bloodletting. The size of the needle varies depending on the tissue to be treated and the body habitus of the patient. No injectate is used. Care with the size of needle is especially important when needling around the chest/neck to prevent a pneumothorax. The technique of needling varies depending on whether a muscle is to be activated, fascia released, scar released, or skin crease/tethering to be released. A detailed knowledge of anatomy and training in myoActivation® is essential to safely and effectively needle soft tissues to relieve myofascial dysfunction. The myoActivation® clinician also needs to assess needle safety for any patient with a current or past intravenous drug use and associated body scars.

Release of scars: The myoActivation® needling technique for release of scars involves the sequential insertion of a 30 g hollow-bore needle in the line of the scar and in any areas of densification around the scar, performing multiple perforations approximately 3 mm apart. Patients often report a “biting sensation” with needling, which occurs even if the scar has been treated with appropriately timed topical anesthetic or pre-needling vapocoolant spray.

Activation of muscles in sustained contraction: The myoActivation® technique for activation of muscles in sustained contraction involves sequential palpation of the muscle to find the most painful point (the active trigger in the muscle). This is best done by palpating bilateral muscle groups simultaneously to help the patient and the myoActivation® clinician determine the most palpable pain point. A 25-30 gauge hollow-bore cutting-tip needle is quickly inserted into that trigger point and immediately removed. If successful, this will be associated with a release of that muscle and may be associated with a muscle twitch (the twitch response), but a twitch response is not mandatory for a successful activation of a muscle in sustained contraction. Repeat palpation at the same site will not be painful if the muscle has been successfully activated. Needling may need to be repeated if there is another palpable pain point in the same muscle group at a different site.

Release of fascial tension: The technique of needling to release fascial tension varies depending on the type of fascial tension that is observed on inspection or on the palpation findings in the region of the most restricted or painful BASE test. If there is a skin crease or skin tethering, these lines of tension are released in a similar technique as indicated above for a scar. If there is a palpable painful density in the fascia, then this is released with three to five quick insertions to resolve that palpable pain and density.

Trauma informed care: Trauma-informed care requires that healthcare teams have a complete picture of a patient’s past and present life situation in order to provide effective, healing healthcare services. A trauma-informed approach requires that the myoActivation® clinician place the patient at the center of the myoActivation® process and unravel an individual's pain and suffering in a manner that is most appropriate for them. Establishing the TiLT in a compassionate way is often the first part of this process. myoActivation® treatment needs to be applied with a trauma-informed approach that supports the patient’s emotional well-being. It has been observed by myoActivation® clinicians that when traumatically injured soft tissues are needled, some patients may recall past events or experience emotional releases. These experiences are very variable but range from mild reflections to vivid recall. These experiences may also evoke memories of associated scents and sounds, emotional awareness, and crying. The patient is usually surprised by these responses to needling and may need time to work through the experience. If strong emotional responses are anticipated, myoActivation® sessions can be delayed while the patient prepares for the treatment and organizes for psychological support. People with lived experience of chronic pain often understand how their emotions and injury history relate to their pain. In the process of unravelling the myofascial components of chronic pain with myoActivation® needling, the emotional experience associated with relief of tension in the body often improves; hence, myoActivation® applied in a trauma-informed manner can play a unique role in holistic healing from chronic pain.

Catenated cycles: Catenated cycles are repeated sequences of BASE testing, palpation, and needling that occur in each myoActivation® session to unravel the multiple sources of myofascial dysfunction. There may be one to six catenated cycles in each myoActivation® session. Each cycle usually identifies the next new and different most painful or restrictive BASE test resulting in a new region for inspection, palpation, and treatment. Catenated cycles demonstrate to the clinician some or all of the following: visible changes in patient movement, increase in joint range, greater range of motion, increase in speed of movement, increase in ease, smoothness, or fluidity of movement, and changes in reported pain with each movement.

For the patient, catenated cycles will demonstrate some or all of the following subjective changes in post-treatment movement: reduction in overall perceived pain at rest and/or movement, reduction or a diffusion in the area of pain, shift in pain location, perception of pain only at end range rather than throughout range, or a different pain focus altogether at a different location that only becomes perceptible when the initial painful site has been treated. Another advantage of the catenated cycles is that the patient has to get up and move after each treatment, which distracts from any discomfort resulting from the treatment process.

Deciding factors on when to stop in any one myoActivation® session: It is optimal to end sessions at a successful endpoint. These might include: resolution of pain, reduction in pain, improved flexibility, increased fluidity of movement, positive postural changes, or a change in the weight distribution of the feet to being more grounded (even plantar weight distribution). Otherwise, the decision during treatment to stop further needle insertions is a clinical judgement that is dictated primarily by the patient‘s ability to tolerate the procedure. Fatigue, feeling overwhelmed, and emotional responses are not uncommon, especially during the first treatment session. An important principle is not to do too much at each session, especially the first one. When the origins of the targeted MFD are from a traumatic event (e.g., abuse, assault, major injury), the patient's readiness for needling needs to be carefully assessed in a trauma-informed approach as outlined above. Sometimes, only one needle insertion can be performed or only one site treated, rather than doing more than one catenated cycle in one session. Fewer needle insertions per session are recommended for a patient with a complex trauma history, heightened arousal, and poor capacity to regulate their emotions.

Aftercare Instructions

Instructions following treatment are directed to promote recovery of treated tissues and prevent symptom regression. Patients are advised to move regularly, with frequent changes in posture (every 10-15 minutes) while awake in the first 24-48 hours after each myoActivation® session. They are also advised to avoid myofascial loading, repetitive exertion, and prolonged postures for 5 days. After this time, they can return to or start a graduate activity. The post-treatment response will be an individualized experience for each patient. Multiple factors will govern the outcome resulting from treatment, including the degree of pre-treatment sedentary activity, physical demands in the workplace, nutrition, patient age, genetically determined responsiveness of soft tissues, and psychosocial factors related to chronic pain.

Number of myoActivation® Sessions Needed to Unravel Multiple Sources of Myofascial Dysfunction

It is optimal to schedule two to three sessions, one or two weeks apart, to minimize the need to do too much at each individual myoActivation® session, minimize discomfort following treatment, and to help determine responsiveness. After three sessions, the myoActivation® clinician can usually determine if there is sufficient positive response to continue. Serial change is observed in positive responders, who usually experience less pain, so that activities of daily living have improved, BASE tests serially improve in terms of the number that can be achieved without discomfort, and in their range of motion. There is a wide range in the number of sessions required for positive responders, but it usually requires two to eight sessions to achieve an improved range of motion in all BASE tests and resolution of chronic pain. This decision to stop myoActivation® sessions is based on the participant's response to needling, changes to the BASE tests, and the patient's report of improvement in pain and ability to perform activities of daily living.

Clinically Observed Benefits of myoActivation®

After treatment, the patient may observe changes in their pain, such as a reduction in intensity or a shift to a different area. They might also find that the pain no longer arises during movements that previously triggered it. Movements are easier to complete and are more fluid in their execution. Patients may notice that they feel more balanced in the weight distribution between their feet.

Appendix 2: complex pain service recommendations for myofascial component to pain

Supplements

Magnesium bisglycinate (dosed as elemental magnesium)

- Recommendation level 1: Increase gradually to 300 mg twice a day (for weight <40 kg).

- Recommendation level 2: Increase gradually to 400 mg twice a day (for weight >40 kg).

- Recommendation level 3: Increase gradually to 600 mg twice a day (for weight >60 kg).

Vitamin K2 - D3 (comes as a combination or can be taken separately)

- Vitamin K2 - 100-120 mcg/Vitamin D3 - 1000 units by mouth twice a day (for weight <50 kg).

- Vitamin K2 - 200-240 mcg/Vitamin D3 - 2000 units by mouth twice a day (for weight >50 kg).

Omega 3

- 1 capsule (~1000 mg of EPA and DHA combined) by mouth once a day.

Select the product with the highest DHA and EPA concentrations.

Mind and Body

- Daily paced physical therapy and gentle muscle stretches, supervised by a physiotherapist.

- Weekly massage by a registered massage therapist (RMT).

- Mind-body techniques to help with pain, supervised by a psychologist/counsellor.

- Healthy nutrition.

- Practice healthy sleep habits (sleep hygiene).
